# Early identification of children with Attention-Deficit/Hyperactivity Disorder (ADHD)

**DOI:** 10.1371/journal.pdig.0000620

**Published:** 2024-11-07

**Authors:** Yang S. Liu, Fernanda Talarico, Dan Metes, Yipeng Song, Mengzhe Wang, Lawrence Kiyang, Dori Wearmouth, Shelly Vik, Yifeng Wei, Yanbo Zhang, Jake Hayward, Ghalib Ahmed, Ashley Gaskin, Russell Greiner, Andrew Greenshaw, Alex Alexander, Magdalena Janus, Bo Cao

**Affiliations:** 1 Department of Psychiatry, Faculty of Medicine and Dentistry, University of Alberta, Edmonton, Alberta, Canada; 2 Ministry of Health, Government of Alberta, Edmonton, Alberta, Canada; 3 Alberta Health Services, Edmonton, Alberta, Canada; 4 Department of Emergency Medicine, Faculty of Medicine and Dentistry, University of Alberta, Canada; 5 Family Medicine, Faculty of Medicine and Dentistry, University of Alberta, Canada; 6 Offord Centre for Child Studies, Department of Psychiatry and Behavioural Neurosciences, McMaster University, Hamilton, Ontario, Canada; 7 Department of Computing Science, University of Alberta, Edmonton, Alberta, Canada; 8 Alberta Machine Intelligence Institute (Amii), Edmonton, Alberta, Canada; Wake Forest University School of Medicine, UNITED STATES OF AMERICA

## Abstract

Signs and symptoms of Attention-Deficit/Hyperactivity Disorder (ADHD) are present at preschool ages and often not identified for early intervention. We aimed to use machine learning to detect ADHD early among kindergarten-aged children using population-level administrative health data and a childhood developmental vulnerability surveillance tool: Early Development Instrument (EDI). The study cohort consists of 23,494 children born in Alberta, Canada, who attended kindergarten in 2016 without a diagnosis of ADHD. In a four-year follow-up period, 1,680 children were later identified with ADHD using case definition. We trained and tested machine learning models to predict ADHD prospectively. The best-performing model using administrative and EDI data could reliably predict ADHD and achieved an Area Under the Curve (AUC) of 0.811 during cross-validation. Key predictive factors included EDI subdomain scores, sex, and socioeconomic status. Our findings suggest that machine learning algorithms that use population-level surveillance data could be a valuable tool for early identification of ADHD.

## Author summary

Many children exhibit symptoms of Attention-Deficit/Hyperactivity Disorder (ADHD) at a young age, but it is often diagnosed at a later stage. This delay in diagnosis can deprive children of the necessary support that they require. To address this issue, we conducted a study to develop a model that could predict ADHD in kindergarteners. We analyzed various information readily available for this age group in 2016, including health records, demographics, and teacher-rated developmental assessments. We then followed these children for four years to evaluate the accuracy of our model in predicting their later ADHD diagnosis. Our findings were promising, particularly when we used all the available data. The scores from developmental assessments were a significant factor in predicting the diagnosis accurately, along with other health and demographic factors. Our results suggest that machine learning could be an effective tool in helping parents, teachers, and doctors identify children with ADHD earlier, leading to better and more timely support.

## Introduction

ADHD (Attention-Deficit / Hyperactivity Disorder) is characterized by developmentally inappropriate, persistent, and pervasive inattention and/or hyperactivity-impulsivity that interferes with daily functioning at home, school, or work [1,2]. Based on a review of 175 studies up to the year 2013, the prevalence of ADHD in children aged 18 and under is estimated to be 7.2% [3] and is increasing [4]. ADHD is associated with emotional dysregulation [5], neuropsychological dysfunction [6], poor social relationships and cognitive skills [7], academic underachievement [8], risky sexual behavior, early pregnancy [9], and criminal activities [10,11]. In turn, the economic impact of ADHD is substantial, with disease-associated costs estimated to be $74 billion and $6 to $11 billion annually in the United States and Canada, respectively, due to losses in productivity [1].

Early interventions in preschool [12] and school-aged [13,14] children, such as behavioral training and stimulant medications, are effective in curbing downstream negative consequences of untreated ADHD. However, diagnosis of ADHD in the preschool years is challenging, delaying early intervention in most cases. For example, in 2016, of 6.1 million ADHD cases diagnosed before 18 years of age in the United States, only 2–6% were diagnosed before four years of age [15], and over half were diagnosed between 12 and 17 years [14–17]. Delayed diagnosis is more prevalent in girls [18]. Factors contributing to delayed diagnosis may include a lack of awareness of ADHD signs/symptoms in parents and teachers. Early identification of children with a heightened risk of ADHD at a young age may raise parents’ awareness and encourage parents to seek clinical diagnostic clarification, and promote early intervention.

However, clinical diagnoses are often not directly captured in population-level data, and the risk of ADHD can only be estimated. One source of identifying probable ADHD cases is administrative health data. In Canada, the health care system is publicly funded, universally available, and administered at the province/territory level. Administrative health data is being collected routinely and used widely for population health surveillance and holds the potential to estimate the risk of clinical diagnoses using case definitions, including ADHD. The validity of using case definitions of ADHD to approximate the clinical diagnosis and population-level prevalence of ADHD was previously explored, yielding high confidence using International Classification of Disease (ICD) codes from physician claims, ambulatory records as well as drug dispensation history [19–22].

In addition to administrative health data, cross-sector data such as population surveillance within the educational system are also routinely collected and may facilitate identifying and estimating ADHD risk. One population-level surveillance tool widely used in the education sector internationally is the Early Development Instrument (EDI) [23,24]. EDI assesses developmental health by identifying children’s vulnerability to poor developmental outcomes based on teacher-completed questionnaires. It provides information about kindergarten-aged children’s (4 to 6 years old) ability to meet age-appropriate developmental expectations shaped by their experiences in the first five years of life [25]. The questionnaire comprises 103 questions involving five domains, including physical health and well-being, social competence, emotional maturity, language, and cognitive development, as well as communication skills and general knowledge [23]. The five domains consist of 16 subdomains, including physical readiness for the school day, physical independence, gross and fine motor skills, overall social competence, responsibility and respect, approaches to learning, readiness to explore new things, prosocial and helping behavior, anxious and fearful behavior, aggressive behavior, hyperactivity and inattentive behavior, basic literacy, interest literacy/numeracy and memory, advanced literacy, basic numeracy, and communication and general knowledge [26,27]. The EDI also contains parent-reported and teacher-recorded medical and developmental diagnoses, including parent-reported formal diagnosis of ADHD [28]. Cross-linkage of data sets across the health and education sectors provides an enriched context for interdisciplinary research focussed on identifying risk factors of developmental disorders and developing data-driven, high-performance health risk predictive models [29].

The literature reported a wide range of risk factors for ADHD, including demographic factors such as family size, low socio-economic status [30] and health history factors such as asthma [31], early exposure to antibiotics [32], increased health utilization [33], and prenatal maternal health [34]. The risk factors for ADHD published in the literature were typically investigated using conventional statistical analysis methods (e.g., [30,32,35]) that concentrated on the data description; the models’ performance and generalizability were not the main focus. Machine learning (ML), which goes beyond conventional statistical analysis, uses sophisticated algorithms to construct predictive models to make precise individual-level forecasts that may be extrapolated to real-world contexts. For instance, ML models are created using training data, and a hold-out test set is used to assess how well they perform in the real world when forecasting the future [36]. The underlying causes of a successful predictive model may provide data on the likelihood of ADHD risk at the individual level. Studies of ML-based mental health prediction using large-scale data have been growing rapidly over the past few years, including successful prediction of a wide range of disorders such as depression [37–39], opioid use disorder [29,40,41], and post-traumatic stress [42]. ADHD prediction using ML has been pioneered using neuroimaging data (for a review, see [43]). Only a few population-scale studies used ML-based ADHD predictions [44,45].

Our research goal was to develop a high-performing predictive model for identifying individuals with childhood ADHD in a four-year follow-up window by applying ML algorithms to population-level administrative health data cross-linked with EDI. We also evaluated the contributing predictive risk factors. This novel approach may facilitate forecasting the elevated risk of future ADHD in the real world.

## Methods

### Data sources

The 2016 EDI data were collected between February and March 2016 and provided by the Ministry of Education, Alberta, Canada. The EDI implementation was offered to all publicly funded schools; however, unlike in other Canadian jurisdictions, opting out was possible for the district, school, and individual families.

In addition to the EDI data, administrative health datasets used in this study included the Alberta Health Care Insurance Plan (AHCIP) Physician Claims, National Ambulatory Care Reporting System (NACRS), Discharge Abstract Database (DAD), the Alberta Health Care Insurance Plan (AHCIP) Population Registry Database, Alberta Pharmaceutical Information Network (PIN) database and Alberta Human Services Drug Supplement Plan database (AHSDSP), Alberta Notice of Birth database, Statistics Canada Census Data (2016). These datasets contain children’s health utilization history, prenatal records, and demographics.

The EDI dataset was matched with the health administrative datasets from the Ministry of Health, Government of Alberta (Alberta Health) based on identifiable information (i.e., name, biological sex at birth, date of birth) and unique provincial health number. All predictive variables except demographics were developed based on a 3-year historical window before enrollment. Personally identifiable information was used for data linkage only. The cross-linked dataset was prepared and anonymized at the Ministry of Health before the researcher’s access for data analysis.

This study was approved by the ethics committee at the University of Alberta (Pro00104650). Informed consents were waived due to minimal risks of secondary analysis, where the Ministry of Health already anonymized the cross-linked data before analysis.

### Sample derivation

**Fig 1:**
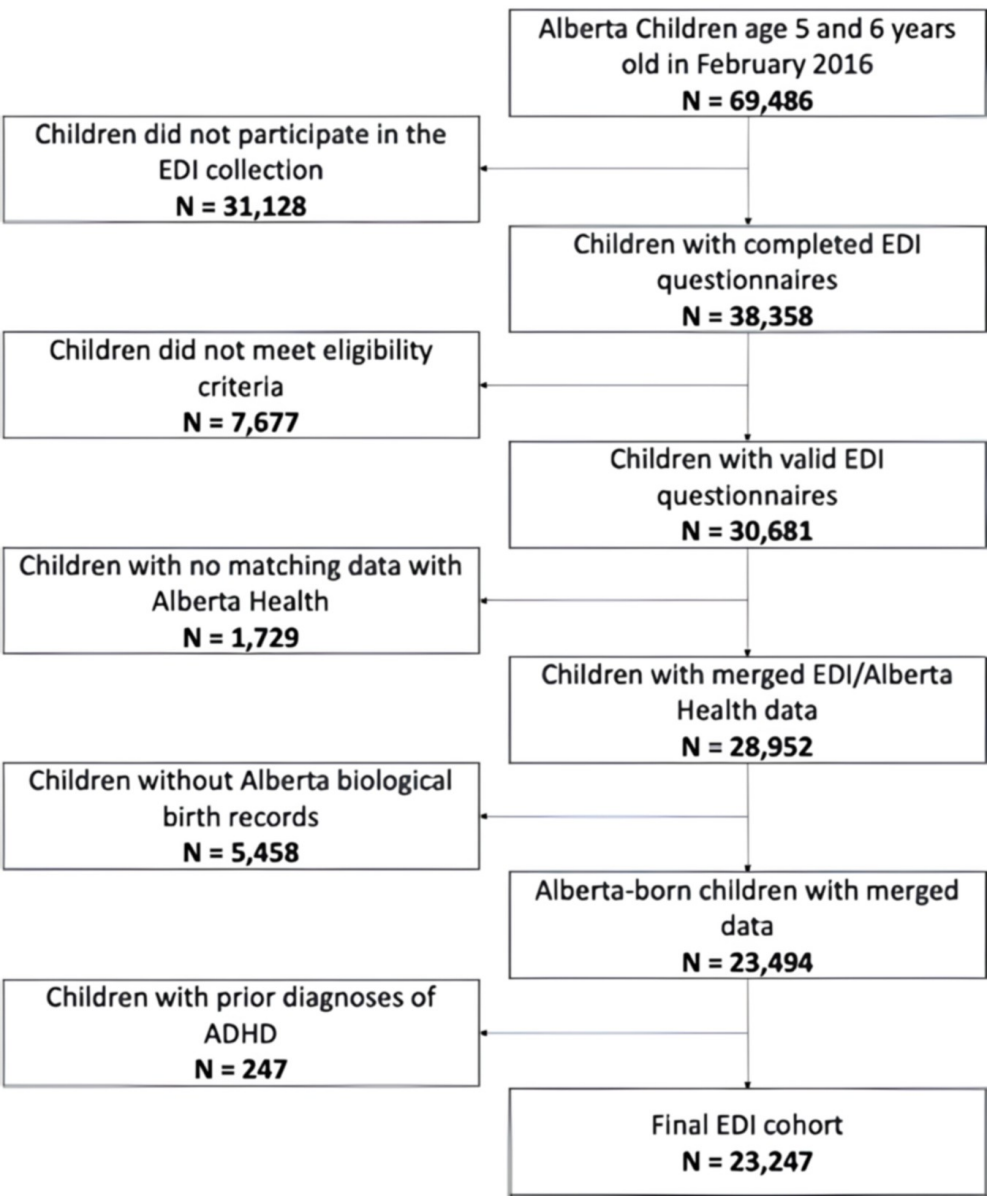
Study Cohort Flow Chart. A flow chart illustrating the derivation of the final EDI cohort.

In Alberta, there were 69,486 children between the ages of five and six in 2016. Of those, 38,358 (55.2%) completed the EDI questionnaire. The reasons that EDI data was not collected for all children aged five and six included: homeschooling, living in remote areas, or school authority and family opt-outs. We first applied exclusion criteria to the EDI dataset, including removing children with missing data, those who attended less than 30 days in the classroom, and a lack of parental or guardian consent records, resulting in the exclusion of 7,677 children. After linking the EDI data with the health administrative data, more children (7,187) were excluded due to mismatch, resulting from either a child not having an Alberta biological birth record (5,458) or the birth record did not match the information in the health administrative data (1,729) (Fig 1).

To ensure the model predicts ADHD prospectively, children with prior ADHD diagnoses before March 31^st^, 2016 (N = 247) were further eliminated from the remaining cohort of 23,494 based on administrative health case definition and EDI. The final cohort for analysis included 23,247 children, with a mean age of 5.68 (SD = 0.33), 48.0% female, 52.0% male, where 8.3% of the cohort belongs to Socioeconomic subsidy group [i.e., Aboriginal, subsidy, welfare]. All predictive variables developed for this dataset were collected before Marth 31^st^, 2016. Based on our case definition (See S1 Table in supporting information), the cohort’s age 4–6 ADHD prevalence rate is 1.1%, which is in line with Canadian reports of 0.8%, 2.0%, and 2.1% prevalence rates for the age group of 5 to 9 years old in Ontario, Nova Scotia, and Quebec, respectively [4]. During the follow-up period, 1,680 kids (7.2%) were found to have case-defined ADHD. See Table 1 for descriptive statistics for ADHD and non-ADHD groups.

**Table 1:** Dataset variable summary.

	**ADHD (n = 1,680)**			**No ADHD (n = 21,567)**			
**Continuous Variables**	**mean**	**std**	**range**	**mean**	**std**	**range**	**t**
Years with asthma	0.33	0.91	0.00–3.00	0.23	0.76	0.00–3.00	5.35*
Years with COPD	0.01	0.13	0.00–3.00	0.01	0.14	0.00–3.00	-0.37
High health utilization^a^ (Number of years)	0.14	0.46	0.00–3.00	0.07	0.32	0.00–3.00	8.30*
Past mental health records of the child (Number of years)	0.58	0.87	0.00–3.00	0.21	0.55	0.00–3.00	25.65*
Years on Human Service Drug Benefit Plan	0.22	0.67	0.00–3.00	0.15	0.55	0.00–3.00	5.36*
≥30% of owner income spent on housing (%)	15.38	3.75	0.00–29.60	15.6	4.11	0.00–29.60	-2.15*
Living in rented dwellings (%)	24.08	13.26	0.00–78.58	23.48	13.54	0.00–79.33	1.73
≥30% of renter income spent on housing (%)	34.88	6.86	0.00–64.60	34.24	7.29	0.00–64.60	3.47*
Living at the same address as 5 years ago (%)	53.7	11.95	19.65–78.11	54.1	12.64	19.65–81.09	-1.25
Not speaking English or French (%)	1.5	1.53	0.00–8.35	1.68	1.7	0.00–8.35	-4.14*
Immigrants arriving within the last 5 Years (%)	5.48	3.87	0.00–20.13	5.75	4.21	0.00–20.66	-2.48*
Percentage of individuals with postsecondary education	64.41	11.32	32.46–87.60	64.08	11.26	24.58–87.60	1.16
Lone parent families (%)	14.66	5.11	5.44–43.28	14.33	5.15	5.44–51.08	2.47*
Average household size	2.71	0.36	1.50–4.50	2.74	0.39	1.50–5.00	-3.16*
Families with low after-tax income (%)	8.89	4.04	1.60–32.00	8.82	4.09	1.60–32.00	0.64
Age	5.69	0.34	4.89–6.88	5.67	0.33	4.55–7.13	1.97
Communication skills and general knowledge	1.86	0.86	1.00–3.00	2.22	0.86	1.00–3.00	-16.48*
Prosocial and helping behavior	1.7	0.8	1.00–3.00	2.07	0.83	1.00–3.00	-17.51*
Anxious and fearful behavior (R)	1.23	0.51	1.00–3.00	1.14	0.41	1.00–3.00	7.85*
Aggressive behavior (R)	1.59	0.83	1.00–3.00	1.16	0.49	1.00–3.00	32.31*
Hyperactive and inattentive behavior (R)	2.05	0.9	1.00–3.00	1.32	0.65	1.00–3.00	42.59*
Basic literacy	2.24	0.83	1.00–3.00	2.55	0.7	1.00–3.00	-17.23*
Interest in literacy/numeracy and memory	2.32	0.84	1.00–3.00	2.65	0.67	1.00–3.00	-18.65*
Advanced literacy	2.13	0.92	1.00–3.00	2.5	0.79	1.00–3.00	-18.52*
Basic numeracy	2.25	0.9	1.00–3.00	2.57	0.75	1.00–3.00	-16.66*
Physical readiness for school day (R)	1.15	0.53	1.00–3.00	1.07	0.36	1.00–3.00	8.69*
Physical independence	2.42	0.91	1.00–3.00	2.77	0.64	1.00–3.00	-20.95*
Gross & fine motor skills	1.89	0.87	1.00–3.00	2.28	0.86	1.00–3.00	-18.12*
Overall social competence	1.88	0.71	1.00–3.00	2.43	0.65	1.00–3.00	-32.91*
Responsibility and respect	2.27	0.78	1.00–3.00	2.78	0.51	1.00–3.00	-37.58*
Approaches to learning	1.98	0.75	1.00–3.00	2.6	0.63	1.00–3.00	-38.77*
Readiness to explore new things	2.66	0.57	1.00–3.00	2.81	0.45	1.00–3.00	-13.02*
**Binary Variables**	**%Yes**	**%No**	**range**	**%Yes**	**%No**	**range**	χ^2^
Mother’s alcohol use status	4.94	95.1	0.00–1.00	2.93	97.1	0.00–1.00	20.45*
Total doctor visits >15	33.5	66.5	0.00–1.00	23.1	76.9	0.00–1.00	99.05*
Emergency department visits > = 4	18.8	81.2	0.00–1.00	12.7	87.2	0.00–1.00	48.17*
Inpatient hospital days> = 1	5.8	94.2	0.00–1.00	3.7	96.3	0.00–1.00	18.32*
Total public health cost > = 5K	20.2	79.8	0.00–1.00	10.5	89.5	0.00–1.00	146.00*
APGARS 5 score > = 8	94.7	5.3	0.00–1.00	96.4	3.6	0.00–1.00	12.48*
Mother has diabetes	5.2	94.8	0.00–1.00	5.6	94.4	0.00–1.00	0.42
Mother > = 4 pregnancies	14	86	0.00–1.00	13.5	86.5	0.00–1.00	0.24
At birth >2 children at home	3.7	96.3	0.00–1.00	4.6	95.4	0.00–1.00	3.03
Mother has hypertension	6.5	93.5	0.00–1.00	5	95	0.00–1.00	6.60*
Communicate adequately in the first language	90.1	9.9	0.00–1.00	92.4	7.6	0.00–1.00	10.62*
Mother’s drug use	4.2	95.8	0.00–1.00	1.7	98.3	0.00–1.00	52.62*
English/French as a second language	4.9	95.1	0.00–1.00	1.7	98.3	0.00–1.00	100.68*
Mother is taking folate	95.2	4.8	0.00–1.00	96	4	0.00–1.00	1.97
Mother has mental health issues at childbirth	14.6	85.4	0.00–1.00	7.6	92.4	0.00–1.00	103.8*
Phototherapy at birth	2.6	97.4	0.00–1.00	2.7	97.3	0.00–1.00	0.03
Preterm birth	10.1	89.9	0.00–1.00	6.8	93.2	0.00–1.00	225.22*
Repeat school grade	5.5	94.5	0.00–1.00	2.8	97.2	0.00–1.00	38.59*
Mother’s smoking status	15.1	84.9	0.00–1.00	8.9	91.1	0.00–1.00	69.36*
Special need status	8.3	91.7	0.00–1.00	1.8	98.2	0.00–1.00	293.2*
Prenatal visits > = 9	73	27	0.00–1.00	6.8	93.2	0.00–1.00	5.97*
Breastfeeding	95.2	4.8	0.00–1.00	96.5	3.5	0.00–1.00	7.21*
Biological sex at birth (female)	26	74	0.00–1.00	50.1	49.9	0.00–1.00	361.33*
Chronic disease status (2015–2016)	25.8	74.2	0.00–1.00	14.9	85.1	0.00–1.00	138.69*
Socioeconomic/Subsidy Status (2015–2016)	11.4	88.6	0.00–1.00	7.8	92.2	0.00–1.00	26.99*

All variables presented were collected before Marth 31st, 2016. T-tests were used to compare groups using numerical measures. Two by two χ2 tests were used to compare groups with binary measures. * denotes p < 0.05 after a false discovery rate (FDR) correction at α = 0.05. R in parentheses denotes reverse coding of the original EDI score; a higher score is associated with more problem behavior. ^a^ High health utilization indication is assigned if the patient had more than 15 visits to a primary health care physician or 10 or more specialist visits or 10 or more ED visits during a fiscal year.

### Outcome definition

The target outcome is whether an individual has ADHD in a 4-year follow-up window, operationally defined as a binary outcome (1 –ADHD, 0 –No ADHD) based on administrative health data-derived case definition. This included ICD 9 and ICD 10 codes connected to inpatient and outpatient visits, psychiatric and mental health facility outpatient visits, physician claims, or a history of stimulant drug use based on Anatomical Therapeutic Chemical (ATC) Classification drug codes (see S1 Table for ADHD case definition). Incidence of ADHD was noted for the cohort in the 4-year period between March 2016 and March 2020.

### Data analysis

Python 3.6 with the scikit-learn 1.0.1 package was used for data pre-processing and ML analysis. A total of 57 predicting variables or features were used for analysis (Table 1). The raw EDI data include 103 questions to evaluate vulnerability in five developmental domains. EDI features were based on categorical subdomain scores. The subdomain scores rated how well the children met developmental expectations at three levels: 1) Met almost all or all expectations (coded as 3). 2) Met some of the developmental expectations (coded as 2), or 3) Met few/none of the developmental expectations (coded as 1) [26]. We reverse coded the scores of physical readiness for the school day, anxious and fearful behavior, aggressive behavior, and hyperactivity and inattentive behavior, switching scores 1 and 3, so a higher score indicates more problem behavior rather than comporting with developmental expectations. In addition, children not fluent in the language of instruction in class (English/French as a second language, n = 2,986) and children with repeated grades (n = 699) were logged as categorical features. Twenty-six features with categorical responses (e.g., ‘Yes’, ‘No’) were dummy coded with the first redundant level dropped (e.g., “Breastfeeding” is first coded as two separate binary coded columns “Breastfeeding = yes”, “Breastfeeding = no”, both columns contain the same information, thus “Breastfeeding = no” is dropped).

We tested a set of linear and non-linear ML models to explore the combined and individual predictive utility of administrative data and EDI data. We also sought to identify important individual predictive factors driving predictions. For linear models, we included the standard Logistic Regression model and Logistic regression model with regularizations, i.e., Logistic Lasso Regression [46] and Logistic Ridge Regression [47]. We included Gradient Boosting [48] and Random Forest [49] for non-linear models. All models were optimized for the area under the receiver operating characteristic curve (AUC) and evaluated using 10-fold cross-validation (CV) with hyperparameter tuning (see S3 Table for details). During 10-Fold CV, the data were split into 10 equal subsets, where each subset was used to validate a model trained with the remaining subsets. As a common practice to enable performance comparison of multiple ML models based on the same processed data [50], all features were standardized using the StandardScaler function to a mean of 0 and a unit standard deviation after training and testing data splitting.

The optimal model was selected based on a performance using AUC. We ran three additional logistic regression models: 1) Administrative health data only (36 features), 2) EDI data only (23 features), and 3) ADHD symptoms (3 features) as baselines for performance comparison. Age and biological sex at birth were included in all baseline models. For all linear models, frequency-based weight adjustments (class_weight = balanced) were applied to control the class-imbalance effect (1,680 ADHD cases versus 21,567 No ADHD controls). The AUC confidence interval was derived based on 30 times of repeats of the 10-fold CV. Non-overlapping confidence intervals were interpreted as statistically different at p < 0.05.

Due to the computational complexity of the study pipeline, no feature selection algorithm was used during modeling. Feature importance was estimated based on ranked average coefficient values from 100 models using bootstrapping. For each of the 10 trained models during 10-Fold CV, we applied 10 times bootstrapping using randomly selected data, each representing 90% of the sample. Following the identification of important features, we aim to explain further how those features impact ADHD identification in its original unit. Some features were presented in a percentage unit but were standardized to zero mean and unit variance in the ML pipeline; thus, they were not directly interpretable. Additional analysis was conducted by fitting a logistic regression model with balanced weight adjustment to the raw data, with ADHD as the dependent variable and all other variables as independent variables, to extract the odds ratios of independent variables.

## Results

### Model performance

**Fig 2:**
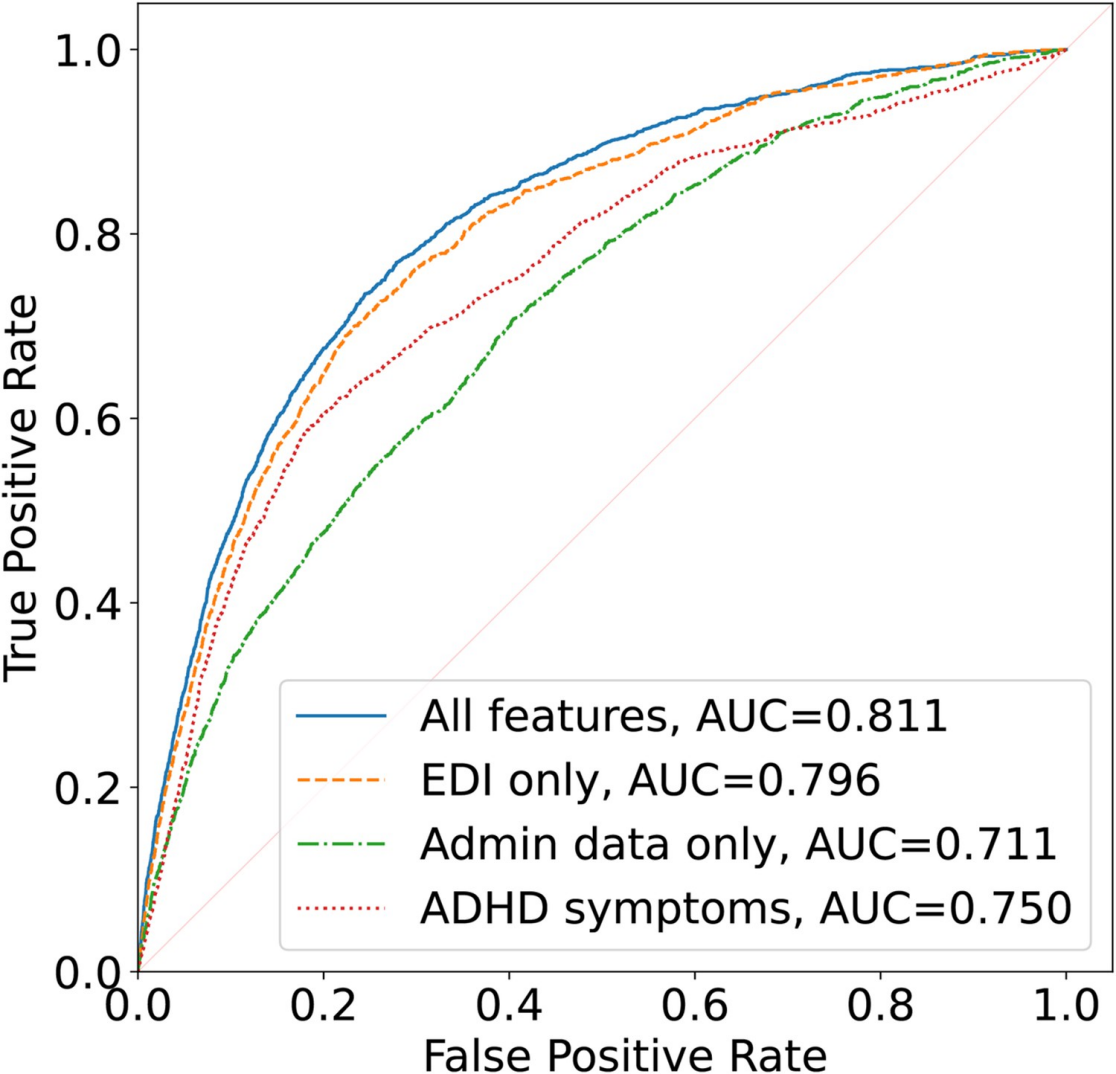
ROC curves. The ROC curves of the best fitting and baseline models. The solid line illustrates the best-fitting model using all available features.

The standard Logistic Regression model without regularisation had a cross-validation AUC of 0.811, representing the best model performance. In contrast, other more complex ML models offered no enhancement of predictive performance (S2 Table). As a result, we concentrated on the outputs of the Logistic Regression to assess the model’s performance in terms of metrics, such as balanced accuracy, and to determine the top 10 predictive features based on feature importance ranking. The Receiver Operating Characteristic (ROC) of the best fitting and baseline models are plotted in Fig 2. The logistic model achieved a cross-validated balanced accuracy of 0.745, with a sensitivity of 0.717 and a specificity of 0.773, presenting a 9.5 percentage points increase in balanced accuracy compared to the model with no EDI features (balanced accuracy = 0.650). Compared to a model using features exclusively from EDI, we found a 0.4 percentage points balanced accuracy difference (balanced accuracy = 0.741). When compared to the ADHD symptoms model using EDI Hyperactive and Inattentive Behaviour score, Sex, and Age as features, we found a 4.3 percentage points balanced accuracy difference (balanced accuracy = 0.702). To facilitate the interpretability of the ML analysis, a logistic regression model with frequency weight adjustment for equal class weight was fitted to the raw data of the entire dataset to generate odds ratios corresponding to the raw data units and FDR adjusted p-value (based on α = 0.05).

### Predictive variables

Table 2 presents the top 10 predictive variables for case-defined ADHD, including four features from EDI. Odds ratios and confidence intervals of odds ratios were calculated based on regular logistic regression fits on raw data with class-balance weight adjustments. This part of the results is a separate analysis from ML prediction of ADHD but presented to facilitate interpretation of the top predictive features identified through ML in its original scale. Approaches to learning help assess how well children work neatly and independently, solve problems, adhere to rules and routines in class, and readily adapt to changes. English/French as a 2^nd^ language indicates whether a child is not a native speaker of the classroom instruction language. Hyperactive and inattentive behavior evaluates the degree to which children show hyperactive behaviors: the ability to concentrate, settle in chosen activities, wait their turn, and think before doing something. Note the scores have been reverse coded, so a higher number indicates more problem behavior in the current study. Overall social competence evaluates the degree to which children have good or excellent overall social development, an ability to get along with other children and to play with various children, cooperative play, and self-confidence.

**Table 2:** Top 10 predictive features for children with ADHD.

Predictive Variable	Odds Ratio	95% CI		p_adjusted_
		Lower	Upper	
**EDI: Approaches to learning**	0.58	0.54	0.62	<0.001
**EDI: English/French as a 2nd language (yes)**	0.35	0.31	0.40	<0.001
**EDI: Hyperactive and inattentive behavior (R)**	1.62	1.54	1.70	<0.001
**EDI and Admin: Biological sex at birth (female)**	0.52	0.49	0.56	<0.001
**EDI: Overall social competence**	0.63	0.59	0.67	<0.001
**Admin: Past mental health records of the child (Number of years)** ^**1**^	1.52	1.44	1.60	<0.001
**Admin: Percentage of individuals with postsecondary education**	1.02	1.01	1.02	<0.001
**Admin: Mother has mental health issues at childbirth (yes)**	1.73	1.57	1.92	<0.001
**Admin: ≥30% of renter income spent on housing (%)**	1.02	1.01	1.02	<0.001
**Admin: Average household size**	1.37	1.17	1.61	<0.001

Odds ratios and confidence intervals of odds ratios were calculated based on regular logistic regression fits on raw data with class-balance weight adjustments. The table follows a descending order of feature importance. Feature importance was ranked based on the magnitude of the bootstrapped coefficients from the ML pipeline. Adjusted p-value based on an FDR correction at α = 0.05. The intercept of the model has a standard estimate of -1.62, with adjusted p = 0.004. CI stands for Confidence Interval. EDI stands for Early Development Instrument. Odds ratios presented are associated with a higher numerical score of the predictor, or “yes” if the predictor is a binary indicator. An odds ratio of less than 1 indicates a reduced risk of ADHD per unit change. An odds ratio greater than 1 indicates an increased risk of ADHD per unit change. R in parentheses (R) denotes reverse coding of the original EDI subdomain score, where a higher score indicates more problem behavior.

^1^Number of years the child was flagged with mental health-related problems between 2013/14, 2014/15, and 2015/16.

A high score on learning strategies, learning English or French as a second language (i.e., not being fluent in the language of instruction), having a female biological sex at birth, and having a high overall social competence is protective against an increased risk of ADHD, according to the multivariate model. A longer history of past mental health records, more hyperactive and inattentive behavior, and a mother’s history of mental health concerns at childbirth were all linked to higher probabilities of ADHD. In addition, demographic data, including a higher percentage of individuals with postsecondary education in the neighborhood, greater than or equal to 30% of income spent on housing, and larger average household size in the neighborhood, were associated with an increased risk of ADHD. The odds ratio of variables in percentage units is associated with per-unit odds change and, thus, needs to be interpreted within this context.

## Discussion

Using cross-linked data from administration health and a population surveillance tool, the EDI, we investigated a ML approach to identify and validate the increased risk of ADHD in kindergarten-aged children in this study. We report an AUC of 0.811 and balanced accuracy of 0.745, demonstrating an increase of 9.5 percentage points when compared against the use of administrative health data alone to predict increased risk of ADHD. The EDI-only model also performed close to the comprehensive model (AUC = 0.796, balanced accuracy = 0.741). The ADHD symptoms model with EDI Hyperactivity and Inattentive Behaviour score, Sex, and Age as features performed in-between the EDI-only model and administrative health data-only model (AUC = 0.750, balanced accuracy = 0.702). The administrative health data alone is underperforming compared to other data but is still better than random prediction (AUC = 0.711, balanced accuracy = 0.650). The result suggests EDI, although designed to be a population surveillance tool for children’s vulnerability, may offer insight to facilitate identifying heightened risk of ADHD. Our results also further contribute to the literature on confirming key risk factors of ADHD that may be used to facilitate early identification and intervention to reduce the harm associated with ADHD.

Early identification of children with a heightened risk of ADHD often starts with parents’ and teachers’ suspicion and is confirmed by physicians later. However, earlier signs of ADHD are often overlooked, even though reliable patterns to identify ADHD may have already emerged. Our study supports that ML application on population-level data may offer a practical tool to identify the overlooked early warning signs and, therefore, raise the red flags for parents, teachers, and physicians, which in turn may translate to early diagnoses and intervention. Although there is currently a lack of comparable studies utilizing similar methods on populational data for children’s ADHD risk screening and a lack of general clinical tools to facilitate early childhood ADHD screening, our model’s performance is comparable to those studies aiming to identify other developmental disorders and childhood ADHD retrospectively. A clinical scale used for screening for autism, the Childhood Autism Rating Scale [51], composed of 24 questions, achieved a sensitivity of 0.71 and specificity of 0.75 in a large sample validation study. In a meta-analysis, pooled sensitivity and specificity of ADHD screening tools ranged from 0.72 to 0.84 [52], with the Conners Abbreviated Symptom Questionnaire reaching a balanced accuracy of 0.83. However, the scales are not designed to identify ADHD in a future time window.

The top four contributing features of our best-performing model are consistently EDI-based features. Higher scores on approaches to learning are protective against ADHD risk (OR = 0.58) and may indicate children with ADHD echo early signs of learning disabilities at kindergarten age [7]. Children learning English or French as a second language have a significantly reduced risk of ADHD (OR = 0.35). To the authors’ knowledge, there’s a lack of empirical findings on the impact of children not fluent in the language of instruction in class on ADHD. However, a lack of English structural skills has been shown to be positively associated with ADHD behavior [53]. Thus, it is likely children not fluent in the language of instruction have a higher risk of ADHD. The reduced risk of ADHD for English and French as a second language children in our model may indicate this is a group of children vulnerable to underdiagnoses of ADHD, a hypothesis that warrants future research. Further, it is not surprising that early observations of hyperactive and inattentive behavior are associated with a 1.62 OR increase for future diagnoses of ADHD, recognized as a primary symptom of ADHD. ADHD is delayed and underdiagnosed in the female population [18]. Thus, females with ADHD were less likely to be diagnosed in our sample of young children. Correspondingly, the female sex reduces the odds of ADHD by half in our model (OR = 0.52). In addition, ADHD children suffer from social incompetency [54], coinciding with our finding that a higher social competency score reduces the ADHD odds by 37% (OR = 0.63).

For a list of notable risk factors from health-data-based predictors, children with more years of past mental health visits, and mother’s poor mental health at birth are associated with largely increased odds of a future ADHD (OR 1.52 per year and 1.73, respectively), consistent with the literature where both children’s health [31–33] and maternal health [34] are risk factors of ADHD. The results cannot inform the underlying cause of increased odds; some plausible explanations may include poor mental health of the child and mother leading to attachment problems, as insecure attachment was high among ADHD children and their mothers [55,56]. However, this finding may inform mental health service providers and policymakers to allocate more mental health resources to parents with mental disorders, such as mothers suffering from post-partum depression or psychosis.

For census-based predictors, the risk of higher ADHD odds increases at OR 1.37 per person for a higher average household size in the neighborhood. This finding is in line with prior reports that larger household sizes coincide with increased childhood adversity, according to Rutter’s indicators [30]. The odds ratio of the percentage of individuals with postsecondary education and greater than or equal to 30% of renter income spent on housing was very low, at 1.02. The average percentage difference between the ADHD and No ADHD groups for those variables was also small in magnitude (e.g., < 1%). Thus, we could not draw a meaningful interpretation based on such small-magnitude effects.

Our results support the hypothesis that cross-linkage of administrative health data and population surveillance data collected by EDI may facilitate accurate individual-level prediction of ADHD, opening opportunities for harm reduction strategies such as promoting awareness of ADHD among teachers, parents, and clinicians and encouraging early access to health care for at-risk children. In recent literature, EDI data has been linked with administrative data records to study medical and social risk factors of non-specific developmental vulnerabilities. One study reported a reasonable concordance between ADHD case definition and EDI records, with a positive predictive value of 61.9% and a negative predictive value of 96.7% [57]. In another study, EDI data were cross-linked with census data to develop behavioral self-regulation profiles of children, showing children with a high-risk profile were more likely to be associated with a subsequent clinical diagnosis of ADHD up to 5 years later [28].

Another insight from the current study results is that administrative health and EDI data both have the potential to facilitate the identification of ADHD even without data crosslinking. Administrative data alone, even though performing subpar to models including EDI data, can be used to perform crude prediction of heightened ADHD risks (AUC = 0.711). It’s also not surprising that EDI data alone perform well in ADHD screening, as parent-reported and school-reported symptom data were often critical to making a diagnosis of ADHD. Importantly, the score on Hyperactive and Inattentive Behaviour also performs well when combined with basic demographic information. The current findings corroborated the literature that symptoms and school performance reports in kindergarten years are predictive for ADHD diagnosis into the school-age years. The current findings set a stage for future follow-up studies to refine predictive modeling algorithms and explore potential real-world applications of big data and ML to inform heightened ADHD risks.

In the current study, we deliberately removed children already diagnosed with ADHD or having a case-defined ADHD label at the time of data collection from our cohort to ensure cross-validation is applied to the prediction of a future label of ADHD and not contaminated with a present label. Thus, the model is trained explicitly for prospective prediction. If this group of children with dual labels (current confirmed ADHD, case-defined ADHD in a 4-year window) are included in training samples, the algorithm may perform at a higher classification accuracy as the ML model has access to more examples of ADHD children to differentiate from non-ADHD children.

Due to a relatively short follow-up window of four years, our data extraction may mislabel a proportion of children with ADHD identified after the follow-up window and falsely label them as having no ADHD in the study. Mislabeling ADHD cases with a delayed diagnosis as No ADHD cases in our model training process may weaken the model’s classification performance in identifying ADHD. Similarly, the model may identify someone who has a higher chance of having ADHD, but in our time frame, the person might not have received a diagnosis. This person is considered a false positive in our model yet could be a true positive case given a more extended time window. Thus, in this sense, false positive predictions from our model could be viewed as a risk indicator for the heightened risk of ADHD. Knowing this, we anticipate future studies with a longer longitudinal follow-up period may yield better classification results and confirm if a model prediction based on a shorter time window could be used for early identification of ADHD.

In addition, the current research focuses on identifying the best prediction model and may offer a limited interpretation of the relative contribution of features that are not prominent predictors of ADHD. The baseline model, including Hyperactive and inattentive behaviour, in addition to Age and Sex, for ADHD prediction performed quite well (AUC = 0.75), which was the strongest predictor.

Another limitation of the study is that the identification of ADHD is based on a case definition derived from administrative health data. This may be a good proxy for true ADHD diagnoses or a heightened risk, but not equivalent to a confirmed clinical diagnosis. The definition of surveillance cases usually has limited specificity but is sensitive and has a high degree of confidence in the identified true cases. For example, some parents may not want children to take psychoactive medication. They may also avoid seeking medical help for various reasons, including considerations such as cost, side effects, social stigma, or believing medication won’t help. Those considerations may result in not visiting a physician or visiting a physician only once without a follow-up, thus not meeting our at least two physician claims criteria and likely missing our criteria for taking ADHD medications. However, when considering the total number of children with case-defined ADHD from 5 to 10 years of age, the identified ADHD rate is higher than expected, at 7.2%. As a comparison, the prevalence rate of ADHD in Ontario, Canada has been estimated to be at 5.6% [58]. The higher prevalence of ADHD identified in our data suggests the case definition used in our study may have introduced more false positive cases, where children with no ADHD risk could have been identified as ADHD cases. Also, the modeling did not extract diagnoses of specific mental disorders and use them as predictive factors and cannot inform if predicted ADHD had comorbid diagnoses. Finally, the modeling pipeline conducted hyperparameter tuning on the full dataset to simplify the computation process, which may cause overfitting and inflate the ML performance. This limitation does not apply to models with no hyperparameter, such as logistic regression.

Future studies should explore further validation of the current study by following up the cohort longer than the 4-year follow-up windows, using clinical diagnoses of ADHD if such data becomes accessible. We also encourage researchers from other geographical regions with cross-linked EDI data to conduct similar analyses. Other areas of future directions include further exploring risk factors of ADHD, building specific models for subtypes of ADHD, ADHD with comorbidity, as well as focused investigation on at-risk populations for delayed diagnoses (e.g., girls).

## Conclusion

In summary, the result of this study suggests that children at risk of ADHD could be identified prospectively at kindergarten age through ML algorithms that use administrative health and population-level surveillance data. The novel application of ML on cross-linked population-level data may have the potential to systematically improve parents’ and clinicians’ awareness of elevated ADHD risks, leading to early diagnosis and, in turn, promoting early intervention to minimize the negative impact of ADHD. We encourage future studies to further validate this approach by using diverse data samples from different regions, and further refining model performance on groups of children vulnerable to delayed diagnoses of ADHD.

## Code availability

The study uses a Python package, dummyML, developed by the authors [59], available from: https://preprints.jmir.org/preprint/65966); code used for analysis is available to access at https://pypi.org/project/end2endML/

## Supporting information

S1 Table ADHD case definition. (DOCX)

S2 Table Model cross-validation performance. (DOCX)

S3 Table Hyperparameter tuning details. (DOCX)
