# Supplementary material for: Early identification of children with Attention-Deficit/Hyperactivity Disorder (ADHD)
Source: PLOS Digit Health. 2024 Nov 7;3(11):e0000620. doi: 10.1371/journal.pdig.0000620 (PMC11542831; doi:10.1371/journal.pdig.0000620)
Supplement: S1 Table — (DOCX) [file pdig.0000620.s001.docx]

**S1 Table. ADHD case definition.**

| Rule | ICD 9 Codes | ICD 10 Codes | Drug Code |
| --- | --- | --- | --- |
| >=1 inpatient visit (1st diagnostic position) OR  >=2 outpatient visits (1st diagnostic position) OR  >=1 outpatient visit in a psychiatric or MH facility OR  >=2 physician claims (1st diagnostic position) OR  Use ADHD medication 2 times in a year | 314, 314.0, 314.00, 314.01, 314.01.a, 314.01.b | F90, F90.0, F90.1, F90.2, F90.8, F90.9 | Stimulants: methylphenidate N06BA04 dexamfetamine N06BA02 amfetamine N06BA01  Non-stimulant: guanfacine C02AC02 atomoxetine N06BA09 clonidine C02AC01 |

Case definition was applied to Alberta residents (as at fiscal year-end); individuals were flagged with ADHD/no ADHD within each year.
