# Supplementary material for: Early identification of children with Attention-Deficit/Hyperactivity Disorder (ADHD)
Source: PLOS Digit Health. 2024 Nov 7;3(11):e0000620. doi: 10.1371/journal.pdig.0000620 (PMC11542831; doi:10.1371/journal.pdig.0000620)
Supplement: S2 Table — (DOCX) [file pdig.0000620.s002.docx]

**S2 Table. Model cross-validation performance.**

|  | **Logistic** | **Lasso** | **Ridge** | **Gradient Boosting** | **Random Forest** | **Baseline**  **(Admin data only)** | **Baseline (EDI only)** | **Baseline**  **(ADHD symptoms)** |
| --- | --- | --- | --- | --- | --- | --- | --- | --- |
| **AUC** | 0.811 | 0.801 | 0.806 | 0.811 | 0.803 | 0.711 | 0.796 | 0.750 |
| **95% CI (Lower)** | 0.810 | 0.800 | 0.806 | 0.810 | 0.800 | 0.709 | 0.796 | 0.749 |
| **95% CI (Upper)** | 0.812 | 0.801 | 0.807 | 0.814 | 0.805 | 0.712 | 0.797 | 0.751 |

AUC stands for the area under the receiver operating characteristic curve. EDI stands for Early Development Instrument. The ADHD symptoms model is a baseline model using EDI Hyperactive and Inattentive Behaviour score, Sex, and Age as predictors.
