# Supplementary material for: Early identification of children with Attention-Deficit/Hyperactivity Disorder (ADHD)
Source: PLOS Digit Health. 2024 Nov 7;3(11):e0000620. doi: 10.1371/journal.pdig.0000620 (PMC11542831; doi:10.1371/journal.pdig.0000620)
Supplement: S3 Table — (DOCX) [file pdig.0000620.s003.docx]

**S3 Table. Hyperparameter tuning details**

| **Model** | **Hyperparameter** | **Hyperparameter Tuning method** | **Function Tuning Settings** |
| --- | --- | --- | --- |
| **Logistic** | None | None |  |
| **Lasso** | Strength of Lasso type penalty | Grid search | A grid of 30 Cs selected from (1e-4 to 1e4), based on best performance on 10-fold CV. |
| **Ridge** | Strength of Ridge type penalty | Grid search | A grid of 30 Cs selected from (1e-4 to 1e4), based on best performance on 10-fold CV. |
| **Gradient Boosting** | Maximum depth of base tree; number of trees | Bayesian optimization | Number of trees (50 to 1500)  Maximum depth (2 to 15)  Learning rate fixed at 0.1. |
| **Random Forest** | Maximum number of features for splitting; number of trees | Bayesian optimization | Number of trees (50 to 1500)  Maximum number of features (From 0.1 to 1.0 at a step of 0.1) |

Hyperparameter tuning was conducted using the entire dataset before cross-validation. The linear model’s hyperparameter tuning was achieved using LogisticRegressionCV function from the Sklearn package version 1.0.1 [1]. Random forest model was fitted using RandomForestClssifier function from Sklearn package version 1.0.1. Gradient Boosting model was fitted using the XGBClassifier function using xgboost package version 1.5.1 [2]. The Bayesian optimization tuning was achieved using Optuna python package version 2.10.0 [3].

**References**

1. Pedregosa, F., Varoquaux, G., Gramfort, A., Michel, V., Thirion, B., Grisel, O., ... & Duchesnay, É. (2011). Scikit-learn: Machine learning in Python. *the Journal of machine Learning research*, *12*, 2825-2830.
2. Chen T, Guestrin C. Xgboost: A scalable tree boosting system. InProceedings of the 22nd acm sigkdd international conference on knowledge discovery and data mining 2016 Aug 13 (pp. 785-794).
3. Akiba T, Sano S, Yanase T, Ohta T, Koyama M. Optuna: A next-generation hyperparameter optimization framework. InProceedings of the 25th ACM SIGKDD international conference on knowledge discovery & data mining 2019 Jul 25 (pp. 2623-2631).
